# Supervisor–group culture, age and the stress–burnout mechanism: a qualitative study of Chinese doctoral students

**DOI:** 10.3389/fpsyg.2026.1794711

**Published:** 2026-03-03

**Authors:** Shuman Xie, Siliang Yu, Lijun Xu

**Affiliations:** 1 Faculty of Education, Sichuan Normal University, Chengdu, China; 2 Faculty of Management, Shinawatra University, Pathum Thani, Thailand

**Keywords:** academic stress and burnout, age and life course, Chinese doctoral students, shi-men, supervisor–group culture

## Abstract

This study examines how supervisor–group culture and age jointly shape Chinese doctoral students’ experiences of academic stress, burnout, self-criticism, and depressive mood in shi-men–based training systems. Drawing on semi-structured online interviews with 28 PhD students from three research-intensive universities in eastern and central China, we use reflexive thematic analysis to trace stress–burnout trajectories across contrasting supervisor–group configurations and two age groups (“younger,” 24–28 years; “older,” 30+ years). We identify two group ecologies—supportive–competitive and laissez-faire–loose—and demonstrate that younger and older students inhabit these ecologies differently. In supportive–competitive groups, younger students often move from stress to comparison-driven self-criticism and muted burnout. Older students, by contrast, describe stress leading to burnout, intensified self-attack, and depressive mood and related distress interpreted as “failing” their life schedule. In laissez-faire—loose groups, by contrast, younger students drift in uncertainty and identity doubt, whereas older students experience silent over-responsibility and resigned low mood. Across both ecologies, supervisors’ responses to age concerns—legitimizing delay, age-based urging, or silence—operate as switches that amplify or buffer these chains. Conceptually, the study extends age-moderated stress–burnout models by theorizing age as a relational life-course project enacted in supervisor–group cultures. It also highlights the need for age-sensitive supervision and policy.

## Introduction

Doctoral students’ mental health has become a major international concern. Systematic reviews estimate that roughly one quarter of PhD students report clinically significant depressive symptoms and about one fifth report clinically significant anxiety (Satinsky et al., 2021; Chi et al., 2023). A recent narrative review similarly concludes that anxiety and related difficulties are widespread and often poorly managed across doctoral programs (Ma et al., 2024). Although newer population-based studies suggest that prevalence may be lower than early “crisis” estimates (Keloharju et al., 2024), doctoral education remains a high-risk context in which academic structures and relationships strongly shape students’ wellbeing.

Research has therefore shifted from documenting prevalence to unpacking mechanisms and contexts. Quantitative studies model how academic stress translates into burnout, self-criticism and depressive symptoms (Jiang et al., 2021; Lin et al., 2025). In Chinese higher education, Li et al. (2023) show that stress and anxiety undermine doctoral performance partly through impaired self-regulated learning and that these associations differ by age. Bai et al. (2025) identify peer comparison, future employment uncertainty and fears of “falling behind” as central anxiety mechanisms. Work on supervision similarly highlights its centrality for doctoral wellbeing: Hazell et al. (2024) show that researchers experience supervision as both a conduit of institutional pressures and a “mirror” of self-worth, while Mavrogalou-Foti et al. (2024) find that uncertainty and mismatch in the supervisory relationship significantly predict depressive symptoms, anxiety and stress. Synthesizing such findings, Acharya and Rajendran (2023) propose a holistic, ecological model in which resources at individual, supervisory, institutional and family levels jointly shape doctoral wellbeing.

The Chinese doctoral system provides a particularly revealing context for examining these dynamics. Doctoral training is typically organized around a single supervisor who leads a research group often described as a shi men, or academic family. Dai and Elliot (2023) show that shi men function as tightly knit communities in which supervisors occupy hierarchical roles and students relate as “brothers” and “sisters,” bound by expectations of loyalty, productivity and reciprocity. These supervisor–group cultures can offer strong academic and emotional support, yet they may also intensify competitive comparison and status anxiety. Recent Chinese studies document high levels of stress, anxiety and burnout linked to publication pressure, competitive group norms and uncertain career prospects (Li et al., 2023; Bai et al., 2025; Ma et al., 2024).

For an international readership, shi men can be situated alongside comparable formations in Western doctoral training, such as PI-led laboratory groups and apprenticeship-like models of doctoral socialization, where learning and academic identity are also organized through participation in a supervisor-centred research collective. These settings similarly combine mentorship, gatekeeping, and peer learning, and are sometimes discussed through notions of academic lineage or “lab families.” At the same time, shi men is distinguished by the explicit use of kinship idioms and the more overtly familialised expectations of reciprocity and hierarchical respect that structure supervisor–peer relations (Dai and Elliot, 2023).

Within this context, age is an important but under-theorized dimension structuring doctoral stress and burnout. Life-course perspectives highlight how increasingly age-diverse doctoral cohorts interpret their progress in relation to social expectations of being “on time” or “late” for milestones such as graduation, stable employment, marriage and parenthood. Feelings of being “out of place” in relation to these norms can precipitate anxiety and withdrawal (Phan, 2024; Yang and Cai, 2022). Qualitative work across settings shows that many older doctoral students juggle employment and caregiving, experience stigma or self-doubt about being “too old,” and are especially sensitive to normative timelines for completion (Templeton, 2021; Šestanović and Siddiqui, 2021). In China, Li et al. (2023) and Bai et al. (2025) likewise report age-related differences in how stress and anxiety relate to performance and in how older students interpret their trajectories, often voicing acute concerns about “falling behind” same-age peers in both career and family domains.

Extending this literature, He and Mu (2025) offer the first explicit theorization of age within stress–burnout processes among Chinese PhD students. Drawing on cognitive appraisal theory, they show that academic stress predicts depression via a sequential pathway through academic burnout and self-criticism, and that age moderates this process. Older students exhibit a weaker direct link between stress and burnout but a stronger association between burnout and self-criticism (He and Mu, 2025). Their moderated mediation model demonstrates that “age matters,” but it conceptualizes age as an individual characteristic and does not examine how age-related meanings and anxieties are produced and negotiated within specific supervisor–group cultures. In particular, it remains unclear how age becomes salient in everyday student interactions, how supervisors respond when students express “age anxiety,” and how these institutionalized interpersonal dynamics reconfigure the stress–burnout–self-criticism–depressive mood chain.

The present study addresses this gap by approaching age as a socially constructed and relational category embedded in supervisor–group cultures in Chinese doctoral education. Drawing on qualitative interviews with Chinese PhD students, we examine two configurations—supportive supervisors within highly competitive groups and laissez-faire supervisors heading loosely organized groups—to explore how age interacts with supervisor–group culture in shaping doctoral stress and burnout. Specifically, we ask how younger and older doctoral students experience and interpret academic stress, burnout, self-criticism and depressive mood (and related distress) within contrasting supervisor–group cultures in China (RQ1); how age-related concerns and “age anxiety” are expressed and negotiated in interactions with supervisors and research-group members (RQ2); and how supervisors’ and groups’ responses to age-related concerns (e.g., legitimizing extensions vs. pressuring timely completion) influence pathways from academic stress to burnout, self-criticism and depressive symptoms (RQ3). By situating He and Mu's (2025) age-moderated stress–burnout mechanism within the institutionalized interpersonal arrangements of Chinese shi men, this study reconceptualizes age as a relational, life-course project that is actively “done” in supervisor–group interactions. It specifies how contrasting supervisor–group ecologies configure stress–burnout pathways for younger and older students and develops context-rich, mechanism-focused accounts of how Chinese doctoral students and their supervisors negotiate age-related pressures, with implications for age-sensitive doctoral supervision and policy within and beyond China.

## Literature review

### Doctoral mental health and stress–burnout mechanisms

The literature on doctoral students’ mental health demonstrates both the scale of psychological distress and the need to understand how it develops. Systematic reviews and population-based studies consistently show elevated levels of stress, burnout, anxiety, and depression among PhD students across countries and disciplines (Satinsky et al., 2021; Estupiñá et al., 2024; Bergvall et al., 2025; Keloharju et al., 2024; Martínez-García et al., 2024). Meta-analyzes and scoping reviews highlight recurrent stressors—precarious funding, publication pressure, unclear expectations, supervisor dependence, and work–life conflict—as key antecedents of distress and attrition (Mahsood et al., 2025; Satinsky et al., 2021). Moving beyond prevalence estimates, process-oriented studies model how academic stress develops into burnout and more severe psychological difficulties. Drawing on stress-process and job-demands–resources frameworks, Hish et al. (2019) show that academic stress predicts burnout, which in turn predicts psychological distress among biomedical PhD students. In the Chinese context, Bai et al. (2025) identify multiple pathways from academic pressure to anxiety via perfectionism, social isolation and career uncertainty. These pathways highlight the complexity of psychological mechanisms in over-expanding doctoral systems. Using semantic network analysis, Chen (2025) further shows that “burnout,” “self-doubt,” “advisor,” and “lab culture” form a tightly connected cluster in Chinese doctoral narratives of wellbeing. Building directly on cognitive appraisal theory, He and Mu (2025) propose a moderated mediation model in which academic stress increases burnout, which heightens self-criticism and, in turn, depression. Age moderates several links in this chain among Chinese PhD students. However, these models still conceptualize stress–burnout mechanisms primarily at the individual level.

### Supervision, research environments and shi men cultures

A second strand of research foregrounds the supervisory relationship and the research environment as pivotal contexts in which stress–burnout mechanisms unfold. Systematic and scoping reviews emphasize that supervision quality, mentoring, and social support are among the most consistent correlates of mental health and life satisfaction among PhD students (Acharya and Rajendran, 2023; Martínez-García et al., 2024). Longitudinal and cross-sectional studies show that satisfaction with mentoring and a sense of belonging in the research group predict higher wellbeing and lower distress (Zhang et al., 2022; Mills et al., 2024). More recent work has linked specific supervisory styles to mental health outcomes. Guo (2025) demonstrates that supervisor–student relationship types are differentially associated with doctoral depression via mediators such as academic self-efficacy and perceived support, whereas Li J. et al. (2025) show that supervisory interactions shape students’ academic habitus and positioning within power-laden fields. Complementary qualitative studies in Hong Kong and Europe reveal that supportive, dialogic supervision fosters flourishing, whereas conflictual or absent supervision undermines confidence, progress, and wellbeing (Almlöv et al., 2025; Li Y. et al., 2025; Martínez-García et al., 2024). Together, this research suggests that supervisory and research-group relationships are central contexts for doctoral stress and coping.

In Chinese higher education, these relational dynamics are institutionalized through shi men culture—a form of academic “family” that organizes doctoral training into a semi-closed community centered on the supervisor. Building on Dai and Elliot’s (2023) communities-of-practice (CoP) conceptualization, we treat shi men not only as a cultural label but as a practice-based formation in which doctoral learning and academic identity are produced through patterned participation in a supervisor-led collective. In this view, what matters is how access to meaningful participation is structured (e.g., opportunities to join projects, receive feedback, and be recognized as a legitimate member), how shared norms and routines are maintained (e.g., expectations for productivity, publishing, and reciprocity), and how peer relations function as sites of evaluation and comparison. A CoP lens is therefore useful for linking supervisor–group culture to doctoral wellbeing: different shi men arrangements can generate different “participation regimes” that either support belonging and development or intensify comparison, uncertainty, and status anxiety. Liu and Dong (2025) further distinguish shi men that resemble “families,” “enterprises,” and “academic teams,” arguing that these variants cultivate distinct aspirations, obligations, and patterns of performance among postgraduate students. Recent work on Chinese STEM laboratories similarly indicates that group supervision practices and lab cultures play a central role in doctoral socialization, setting informal norms for productivity, publishing, and peer competition (Wang et al., 2025). This scholarship underlines that supervision in China is rarely a purely dyadic phenomenon; it is institutionalized through group-based cultures that distribute power, resources, and recognition. At the same time, studies of abusive or overly controlling supervision link such patterns to poorer mental health via thwarted autonomy and weakened professional identity (Yao et al., 2025). Yet we still know little about how different configurations of supervisor behavior and group culture—for example, “supportive supervisor–competitive group” versus “laissez-faire supervisor–loose group”—shape stress–burnout mechanisms.

At the same time, the phenomenon we examine should not be reduced to an “Asian issue.” Group-based doctoral training and PI-centred research groups are common across research-intensive systems, and international studies similarly show that supervisory relationships and research-group climates shape belonging, progress, and distress (Zhang et al., 2022; Mills et al., 2024; Martínez-García et al., 2024). What differs cross-culturally is the cultural grammar through which these arrangements are legitimized and lived. In shi men, supervisory authority and peer relations are often explicitly familialised and moralised through expectations of loyalty, reciprocity, and hierarchical respect (Dai and Elliot, 2023; Liu and Dong, 2025). We therefore treat “culture” not as a background descriptor but as a set of normative meanings that shape how pressure, comparison, and belonging are enacted—and how students interpret distress as personal deficiency versus a relationally produced condition.

### Age, life course and doctoral trajectories

Age and life-course considerations add a further under-theorized layer to this picture. Quantitative studies frequently report age differences in doctoral satisfaction, progress, and mental health. Older or later-stage students often face greater work–life conflict, financial pressure, and fears of losing tuition rights (Estupiñá et al., 2024; Yang and Cai, 2022). In the Chinese context, Li et al. (2023) find age-related differences in how stress and anxiety relate to doctoral performance, suggesting that age structures not only access to resources but also interpretations of academic risk. He and Mu (2025) show that age moderates pathways from stress to burnout, self-criticism, and depression in Chinese PhD students. They point to appraisals such as “time running out” as potential intensifiers of vulnerability. From a qualitative life-course perspective, Phan (2024) illustrates how “delayed success” narratives among Vietnamese international doctoral students interweave academic trajectories with family responsibilities, age norms, and labor-market expectations, shaping decisions about dropout. Research on mature doctoral candidates in other settings indicates that many juggle employment and caregiving, experience stigma or self-doubt about being “too old,” and are especially sensitive to normative timelines for completion (Templeton, 2021; Šestanović and Siddiqui, 2021). Taken together, this work suggests that age is not merely a background demographic variable but part of a broader life-course project through which doctoral students evaluate their trajectories. However, existing studies rarely connect these life-course dynamics explicitly to stress–burnout mechanisms or examine how age identities are negotiated within doctoral teams and supervisory relationships.

### Towards multi-level, relational frameworks

Recent theoretical work increasingly calls for multilevel, relational approaches that integrate these strands. Drawing on conservation of resources and self-determination theory, Acharya and Rajendran's (2023) holistic model conceptualizes doctoral wellbeing as emerging from interactions among personal, relational, and institutional resources, including supervisory support and departmental climate, and work on transformational leadership in higher education similarly highlights how leadership practices shape institutional climates and learning environments (Bohari et al., 2024). Martínez-García et al. (2024) similarly propose an integrative framework in which individual vulnerabilities, relationships with supervisors and peers, and systemic conditions jointly structure PhD wellbeing. In the Chinese context, Chen (2025) shows that “advisor,” “lab,” and “institution” co-occur frequently with emotion-laden terms in doctoral narratives of stress and wellbeing. This pattern suggests that institutional and interpersonal factors are tightly embedded in doctoral students’ subjective experiences. Nonetheless, within this body of work, age is often treated as a control variable or simple moderator rather than as a socially constructed dimension of inequality and expectation enacted within supervisor–group cultures.

Taken together, existing scholarship shows that doctoral stress–burnout pathways are a persistent concern; supervisory relationships and research-group cultures are central to doctoral socialization and wellbeing; Chinese shi-men structures institutionalize these supervisor–group dynamics in distinctive ways; and age and life-course trajectories shape how doctoral students evaluate progress, risk, and “timeliness.” Rather than treating age as a background demographic variable, this body of work points to the importance of examining how age-related meanings and pressures are produced and negotiated within supervisor–group contexts, and how such processes become consequential for stress, burnout, self-criticism, and mood. In the next section, we outline the qualitative design, sampling, and analytic strategy used to examine these processes.

## Method

### Research design

This interview-based qualitative study explored how supervisor–group culture and age jointly shape Chinese doctoral students’ experiences of academic stress, burnout, self-criticism, and depressive mood and psychological distress. Rather than estimating statistical associations, we sought to reconstruct the contextual processes and meanings through which these experiences are produced and made consequential in everyday doctoral life (Maxwell, 2012; Acharya and Rajendran, 2023). Working from an interpretivist–critical realist stance, we treat participants’ accounts as situated interpretations of relational and structural conditions (e.g., shi men hierarchies, age-graded expectations), while recognizing that culture and discourse mediate how such conditions are perceived, negotiated, and narrated (Acharya and Rajendran, 2023; He and Mu, 2025). Accordingly, our claims concern participants’ lived and narrated experiences, not clinical diagnosis.

### Terminology

This qualitative study does not make clinical claims. We use depressive mood to refer to participants’ narrated experiences of persistent low mood, hopelessness, emotional numbing, and related distress, as described in everyday language. Where participants used the term “depression” (or “depressed”), we treat this as an emic label rather than evidence of clinically diagnosed depression. When discussing prior quantitative literature (e.g., He and Mu, 2025), “depression” refers to depression-scale scores (i.e., self-reported depressive symptoms) as operationalized in those studies.

### Context and sampling

Fieldwork took place at three research-intensive universities in eastern and central China that operate supervisor-centered, shi men-based doctoral training systems (Dai and Elliot, 2023), here referred to as Universities A, B, and C. In these institutions, doctoral education is organized around a single supervisor leading a relatively stable research group or academic “family.”

We used purposive, theoretically informed sampling to achieve maximum variation along two axes central to our research questions: age group and supervisor–group configuration. Drawing on He and Mu's (2025) age-moderated stress–burnout model and empirical work on shi men cultures (Dai and Elliot, 2023), we constructed a 2 × 2 sampling matrix with two age groups: “younger” (approximately 24–28 years, typically transitioning directly from prior study) and “older” (30+ years, often with work and/or family responsibilities). We crossed these with two supervisor–group cultures: supportive supervisors within competitive, high-pressure groups and laissez-faire (hands-off) supervisors within loose, weakly integrated groups.

Participants were recruited via doctoral mailing lists, online bulletin boards, and snowball sampling, with clear statements that participation was voluntary and unrelated to assessment. We interviewed 28 students (15 women, 13 men; aged 24–37 years), evenly split between the younger and older groups. Within the 2 × 2 sampling matrix, there were seven participants in each age-by-configuration cell (younger–supportive–competitive, older–supportive–competitive, younger–laissez-faire–loose, older–laissez-faire–loose). Further demographic and configuration details are provided in Appendix 3. Sample size was guided by Malterud et al.'s (2016) notion of “information power.” Given our focused aim, theoretically driven sampling, and rich interview material, a sample in the high 20s was judged adequate for in-depth thematic analysis.

Although we report participants’ gender and discipline in Appendix 3, the study was designed around age group and supervisor–group configuration as the primary sampling and analytic axes, because these dimensions were central to our research questions. We nevertheless recorded gender and disciplinary background because they are plausibly implicated in how age is experienced and narrated in doctoral life—through gendered life-course expectations (e.g., family planning and caregiving responsibilities) and through disciplinary norms around productivity, publication rhythms, and supervisory practices. During coding, we flagged instances where participants explicitly connected age-related concerns to these dimensions and used such observations to contextualize interpretations. However, the sample and study design were not intended to support systematic subgroup comparisons by gender or discipline; we therefore treat these dimensions as contextual descriptors and sensitizing lenses rather than focal analytic categories.

### Data collection

Data were generated through semi-structured, one-to-one interviews conducted via encrypted online videoconferencing between September and November 2025. Semi-structured interviewing allowed us to cover core topics while also leaving space for participants’ own interpretations and examples (Brinkmann and Kvale, 2015). Each interview lasted approximately 40–60 min. All three authors conducted the interviews in Mandarin with participants’ consent for audio recording. All interviews were audio recorded and transcribed verbatim. During transcription, identifying information (names of individuals, supervisors, labs, and departments) was removed or replaced with pseudonyms.

The interview guide was informed by He and Mu's (2025) moderated mediation model of academic stress, burnout, self-criticism, and depression; life-course perspectives on age and doctoral trajectories; and research on shi men and doctoral supervision in China (Dai and Elliot, 2023). It covered participants’ backgrounds; relationships with supervisors; research-group and shi men culture; experiences of stress, burnout, self-criticism, and negative mood; the salience of age and “age anxiety”; supervisors’ and groups’ responses to age-related concerns; and coping strategies and resources. Interviews were conducted in Mandarin to maximize participants’ expressive nuance, and quotations were subsequently translated into English and lightly edited for clarity while preserving meaning. When participants used colloquial terms such as “depression,” we treated these as self-reported experiences of low mood/distress within their narratives rather than as medical diagnoses. Throughout, we prioritized confidentiality by removing identifying details and using pseudonyms. Appendix 1 presents the semi-structured interview guide and example prompts.

### Data analysis

We employed reflexive thematic analysis (Braun and Clarke, 2019, 2022), treating coding and theme development as iterative interpretive work rather than mechanical categorization. Our analysis was sensitized by He and Mu’s (2025) stress → burnout → self-criticism → depressive mood pathway and by multilevel resource frameworks of doctoral wellbeing (Acharya and Rajendran, 2023), while remaining open to inductive patterns—especially regarding how age becomes salient in everyday interactions and how supervisor–group cultures are enacted.

Transcripts were managed in NVivo 14. After repeated reading for familiarization, the first and second authors independently coded a strategically varied subset of six interviews (balanced across age groups and supervisor–group configurations), generating initial semantic and latent codes. The full author team then met to discuss overlaps and discrepancies, refine code definitions, and agree a flexible coding framework. This framework organized codes into nine broad families (e.g., academic stress/burnout; self-criticism and mood; supervisor practices; group norms and shi men culture; age dynamics and responses to age anxiety; coping and resources). The framework functioned as a guide rather than a fixed codebook; codes were added, split, or collapsed as the analysis progressed. Appendix 2 provides an abbreviated coding manual for transparency.

On the basis of codes related to supervisor practices, group norms, and shi men dynamics, we developed an inductive typology of supervisor–group ecologies. “Supportive–competitive” cases combined intensive supervisory involvement and access to resources with tightly knit groups characterized by strong productivity norms and frequent peer comparison; “laissez-faire–loose” cases involved infrequent contact, minimal structured feedback, weak peer integration, and diffuse expectations. Each case was allocated to an ecology through team discussion, with all authors cross-checking the assignment against the full transcript to ensure coherence. These ecologies are analytic ideal types derived from this dataset; other configurations may exist beyond our sample. We then developed themes at two levels: within-case narrative sequences linking age, ecology, and stress–burnout trajectories, and cross-case patterns across the 2 × 2 matrix. Theme refinement involved revisiting transcripts, testing alternative interpretations (including deviant cases), and iterating theme boundaries until the account was coherent and distinct.

### Trustworthiness, reflexivity and ethics

We drew on Tracy's (2010) “big-tent” criteria, aiming for rigor, credibility, and sincerity. Rigor and credibility were supported through theoretically informed sampling, sufficient interview time, systematic analysis, thick contextual description, and attention to deviant cases. We also invited a limited form of member reflections. A short written summary of preliminary themes was shared with six volunteer participants, whose feedback affirmed our interpretations and prompted us to sharpen the contrast between “supportive–competitive” and “laissez-faire–loose” supervisor–group configurations.

Reflexively, all three authors are Chinese scholars working in Chinese higher education. The first author completed doctoral training within a shi men–style research group at a Chinese university and now supervises and teaches in similar settings, providing insider familiarity with supervisor-centered group cultures, age-graded expectations, and the everyday language of “age anxiety.” The second and third authors completed their doctorates at universities outside China, in systems without shi men–based supervision, and subsequently returned to work in Chinese universities. They bring more “outsider” perspectives on shi men while sharing linguistic and broader cultural knowledge with participants. In line with the analytic process described above, the first and second authors led interview-based interpretation and initial coding, while the third author acted as a critical reader of the coding framework and theme development. Throughout the study, we used reflexive memos and regular team discussions to consider how our positionalities and assumptions (for example, about “appropriate” age for doctoral study or about what counts as “supportive” supervision) might shape interviewing, coding, and interpretation (Maxwell, 2012; Braun and Clarke, 2019).

Ethical approval was granted by the Faculty of Education Ethics Committee at the lead university. Participants received written and verbal information about the study’s aims, voluntary nature, data handling, and limits of confidentiality, and they provided written informed consent. We emphasized that supervisors would not know who had taken part and that participation would have no bearing on academic evaluation. When interviews touched on intense distress, the interviewer checked participants’ comfort and, where appropriate, signposted counseling and support services.

## Findings

The analysis generated four interrelated themes that explain how supervisor–group culture and age shape doctoral students’ experiences of stress, burnout, self-criticism, and depressive mood. We first outline the two main supervisor–group configurations and then show how younger and older students inhabit these settings differently. Finally, we examine how supervisors’ responses to age concerns reshape the stress–burnout mechanism. All participant names are pseudonyms, and ages refer to participants’ age at the time of interview. All quotations were translated from Mandarin and lightly edited for clarity.

### Two supervisor–group ecologies

Across universities and disciplines, participants described two contrasting supervisor–group “ecologies”: supportive–competitive groups and laissez-faire–loose groups. These ecologies are inductively derived analytic ideal types developed from this dataset to summarize recurring patterns; they are not intended as an exhaustive classification of doctoral supervision, and other configurations likely exist beyond our sample. While we did not conduct systematic subgroup comparisons, participants’ accounts occasionally suggested that disciplinary norms and gendered life-course expectations shaped how age concerns were articulated and managed.

In supportive–competitive groups, supervisors were present and involved. Students met frequently with them, received detailed feedback, and had access to projects, networks and funding. At the same time, strong norms of productivity and comparison permeated daily life:

*“Our professor is very nice, he reads every draft line by line. But everyone in the group is publishing all the time, so if you do not have a paper this semester you feel you are dragging the team”* (Ying, 27, STEM, younger).

In laissez-faire—loose groups, supervisors were physically and psychologically distant; students reported long gaps between meetings, vague expectations and weak group cohesion:

*“My supervisor trusts us a lot, but basically you manage yourself. Maybe we have a group meeting once a semester and he just says, ‘How’s it going?’”* (Bo, 26, social sciences, younger).

Both ecologies generated stress, but through different mechanisms. Supportive–competitive groups concentrated pressure in dense relational networks and constant comparison; laissez-faire–loose groups produced diffuse stress through under-guidance, uncertainty and isolation. Age shaped how students positioned themselves within these ecologies and how the stress–burnout–self-criticism sequence unfolded.

### “Running against the clock” in supportive–competitive groups

Within supportive–competitive groups, younger and older students faced similar demands but interpreted them through different temporal lenses.

#### Younger students: comparison-driven self-criticism and muted burnout

Younger participants (mid-twenties) often framed high demands as a sign of belonging to a “strong group” and initially welcomed pressure as motivating:

*“I knew this group was famous and stressful. At the beginning, it felt like positive pressure—everyone is running, so I also run”* (Wei, 25, STEM, younger).

Over time, however, constant comparison with high-performing peers fed self-criticism. Students monitored how many papers others had, who had secured grants or job offers, and who received the supervisor’s praise. When they felt they were “behind,” an inner critical voice was triggered:

*“When junior students already publish and I still have nothing, I think maybe I do not deserve this supervisor”* (Lin, 26, humanities, younger).

For many, self-criticism preceded overt burnout. They reported working increasingly long hours to “catch up,” then gradually feeling emotionally depleted, irritable or “numb.” Yet they hesitated to label their state as burnout, suggesting that as young students they were not entitled to speak of exhaustion. The chain they narrated often followed the pattern: stress → self-comparison → self-criticism → emotional exhaustion and disengagement.

#### Older students: compressed timelines and intensified self-attack

Older participants (30+) in supportive–competitive groups faced the same performance norms but overlaid them with compressed life-course timelines. Many had left stable jobs or delayed marriage and childbirth to do the PhD. They interpreted group comparison through a sense of “running against the clock”:

*“My lab mates are 24, 25. If they delay graduation, they are still young. If I delay, maybe I lose the chance for a decent job and also having a second child. The same stress weighs more on me”* (Hui, 33, STEM, older).

Stress from publication and project deadlines translated rapidly into emotional exhaustion. The link from burnout to self-criticism was particularly strong: older students saw slow progress as not only academic failure but also a moral failure toward family and supervisor:

*“I chose this path so late. My parents supported me again, my supervisor gave me a chance, and I am still not good enough. It’s shameful”* (Chen, 35, social sciences, older).

Several reported somatic symptoms alongside low mood and hopelessness. Here, the sequence they described approximated, but intensified, the age-moderated model: stress → burnout → harsh self-criticism → depressive mood and related distress, with age turning ordinary setbacks into existential threats.

### Drifting and over-responsibility in laissez-faire–loose groups

In laissez-faire–loose groups, supervisor distance and weak group cohesion created distinct burnout pathways.

#### Younger students: drifting in a vacuum

Younger students in laissez-faire–loose groups described the early PhD period as marked by “freedom” and lack of structure:

*“He said, ‘I do not like to control you, you are adults.’ At first it felt relaxing, but later it became floating—every day I’m busy with small things but I do not know if I’m moving”* (An, 25, social sciences, younger).

The primary stressor was uncertainty: not knowing what pace of progress was acceptable, how to judge their own work, or when the supervisor might intervene. Many postponed empirical work or writing, then experienced sudden spikes of panic before formal milestones. Burnout emerged as emptiness rather than overwork:

*“Sometimes I sit the whole day in the office and at night I feel I did nothing. It’s like the PhD is passing but I’m not really in it”* (Bo, 26, social sciences, younger).

Identity doubt was tightly entangled with stress. Students interpreted their drifting as evidence they were “not PhD material,” and global self-doubt sometimes appeared before they labeled their feelings as stress or exhaustion. Low mood and withdrawal were common, but often framed as “laziness” or “poor self-discipline” rather than as potential mental-health concerns.

#### Older students: silent over-responsibility

Older students in laissez-faire–loose groups initially seemed to cope well with supervisor distance, drawing on prior work experience. Yet their narratives revealed a heavy sense of silent over-responsibility:

*“Because I’m older and have work experience, the professor assumes I can handle everything. He never checks on my progress. Sometimes I wish he would ask, but I also do not want to look weak”* (Li, 32, humanities, older).

Many were juggling doctoral work with family and financial obligations, studying at night after caregiving. Without strong academic guidance, they worried constantly about “falling behind” without being able to calibrate how serious this was. Burnout took the form of chronic exhaustion and emotional numbing:

*“I feel like a machine. During the day I take care of my child and parents, at night I read, but the ideas do not go in. I just tick boxes”* (Mei, 34, social sciences, older).

For this group, the chain often moved quickly from stress to a resigned mood, bypassing explicit self-attack. Rather than harsh self-criticism, several expressed a quiet hopelessness— “maybe it’s just my fate to be average”—and rarely sought help. Because supervisors did not ask about personal circumstances, age-related pressures (childbearing, employment age limits) remained invisible in the academic setting, reinforcing isolation.

### Supervisors as amplifiers, buffers and silencers of age anxiety

A cross-cutting theme concerned how supervisors and groups responded when age surfaced, and how these responses altered the stress–burnout–self-criticism sequence.

#### Legitimizing delay: partial buffering

Some supervisors responded to age concerns by legitimizing slower progress or extensions and by reframing worth beyond age:

*“I told him I would be 35 when I graduate. He said, ‘You started late, so it’s normal. Your value is not your age but the quality of your work.’ Hearing that, I cried”* (Hui, 33, STEM, older).

Such conversations temporarily weakened the stress–self-criticism link. Students felt seen as persons rather than as delayed timelines and reported reduced shame and more realistic planning. However, where group norms still celebrated early finishers and high publication counts, the buffering effect was fragile: comparison soon reactivated age anxiety.

#### Age-based urging and silence

Other supervisors used age explicitly to justify acceleration:

*“He told me, ‘You are not young. If you do not graduate next year, no one will hire you. I push you now so you will not regret it later.’ I know he means well, but every time I hear this my heart races”* (Zhao, 31, STEM, older).

For some, this functioned as a spur; for others, especially those already exhausted, age-based urging intensified the burnout–self-criticism–depressive mood link. Age became ammunition for an internal voice: “Not only did you miss the deadline, you are wasting your whole life schedule.”

A third pattern, especially in laissez-faire–loose groups, was silence. Many participants had never discussed age with their supervisors. Some had tested the ground with a joke or brief comment, only to be deflected:

*“I joked, ‘Teacher, I’m already old, can I still find a job?’ He laughed and said, ‘Do not overthink, just write your thesis.’ That was the end”* (Mei, 34, social sciences, older).

In these cases, age anxiety remained internal and unprocessed, feeding either self-criticism or resignation. Peer interactions partially filled the gap: some older students found solidarity with other “latecomers,” while others experienced teasing from younger lab mates about being “uncle” or “auntie” still in school. In the absence of explicit norms from supervisors, these peer dynamics could either buffer or intensify age-related shame.

### Re-patterning the stress–burnout mechanism through “doing age”

Across the four age × culture combinations, participants narrated distinct variants of the stress–burnout–self-criticism–depressive mood chain: (1) Younger–supportive–competitive: stress → comparison → self-criticism → burnout, with muted acknowledgement of exhaustion; (2) Older–supportive–competitive: stress → burnout → intensified self-criticism → depressive mood (e.g., persistent low mood and hopelessness), with age turning setbacks into existential threats; (3) Younger–laissez-faire–loose: under-guidance → drifting stress + identity doubt → burnout as emptiness, with global self-doubt sometimes preceding recognition of stress; (4) Older–laissez-faire–loose: multiple responsibilities + lack of support → chronic exhaustion → resigned low mood, often with suppressed or indirect self-criticism.

Supervisory responses to age concerns acted as switches and amplifiers within these chains. Legitimizing delay and reframing worth could loosen the coupling between stress and self-criticism; age-based urging, while occasionally motivating, often strengthened the link between burnout, self-attack and depressive mood for older students; silence allowed age anxiety to grow unchecked, particularly in loose groups where other supports were weak.

Overall, age appears not as a background demographic moderator but as a relational and moral category that is actively “done” in supervisor–group interactions. In the context of Chinese shi men systems, where supervisors and groups are key sites of academic and life-course evaluation, “doing age” is a central mechanism through which structural pressures are converted into burnout and deeper forms of suffering. These findings provide the empirical basis for the discussion section, where we re-situate age-moderated stress–burnout models within institutionalized interpersonal arrangements and consider implications for age-sensitive doctoral supervision and policy.

## Discussion

This study examined how supervisor–group culture and age jointly shape Chinese doctoral students’ experiences of academic stress, burnout, self-criticism and depressive mood in shi-men–style training systems. Building on evidence that PhD students report elevated levels of depressive symptoms and anxiety compared with other highly educated groups (Satinsky et al., 2021; Chi et al., 2023), our analysis moves beyond individual risk factors to show how stress–burnout mechanisms are configured within institutionalized interpersonal relations and life-course expectations.

### Extending age-moderated models into institutional–relational terrain

Quantitatively, He and Mu (2025) showed that academic stress predicted depressive symptoms (depression-scale scores) via burnout and self-criticism. Age moderated several links in this pathway: older Chinese PhD students exhibited stronger burnout–self-criticism–depressive-symptom pathways than younger peers. Our qualitative analysis underscores the importance of age, but suggests that treating it as a static demographic moderator is insufficient. Instead, “age” operates as a relational and moral category that is actively enacted within supervisor–group cultures and broader social timelines.

Across the four age × culture combinations, participants described distinct variants of the stress–burnout chain. Younger students in supportive–competitive groups reported a sequence of stress → comparison → self-criticism → exhaustion, often downplaying burnout because it felt incompatible with being “young and energetic.” Older students in the same groups described stress → burnout → intensified self-attack → depressive mood, interpreting slow progress as a failure of their entire life schedule. In laissez-faire—loose groups, younger students drifted between under-guidance, uncertainty and global self-doubt, whereas older students moved from chronic over-responsibility to resigned low mood, often bypassing explicit self-attack.

These patterns extend He and Mu's (2025) model by showing that age moderates not only the strength of specific paths but also the shape of the mechanism as lived in different ecologies. They resonate with ecological frameworks that conceptualize doctoral wellbeing as emerging from interactions among personal, relational and institutional resources and demands (Acharya and Rajendran, 2023). They also highlight the need to theorize age as a socially produced dimension embedded in those interactions.

### Supervisor–group culture as an active mechanism

Our findings also enrich the rapidly growing literature on supervision and doctoral mental health. Prior work has identified the supervisory relationship as a central predictor of distress and wellbeing (Hazell et al., 2024; Mavrogalou-Foti et al., 2024) and has shown that supervision can function both as a conduit of institutional pressures and as a source of validation (Hazell et al., 2024). We extend this literature by specifying how particular supervisor–group configurations operate as mechanisms that channel stress into different psychological outcomes.

In supportive–competitive groups, supervisors were hands-on, responsive and well resourced, but group norms emphasized constant comparison and high output. This combination resembles what Nagy et al. (2019) describe in biomedical programs where high demands and close evaluation coexist. For some of our participants, such settings fostered engagement and a sense of belonging; for others, particularly older students, heightened comparison turned burnout into moralized self-attack. In laissez-faire—loose groups, supervisors’ distance and ambiguous expectations produced a different risk profile. Identity confusion, under-guidance and isolation became the primary stressors, echoing findings that “uncertain” supervision is associated with poorer mental health (Mavrogalou-Foti et al., 2024).

Importantly, in shi-men contexts, these are not merely individual supervisory styles but features of semi-closed “academic families” (Dai and Elliot, 2023). Our study extends work on shi-men as communities of practice by foregrounding stress, burnout and mood as consequences of how participation, membership, and recognition are organized in these familialised formations. Put differently, a CoP perspective highlights how these ecologies structure differential access to meaningful participation, feedback, and peer comparison—processes through which stress is translated into burnout and depressive mood and related distress. Supervisor–group culture should therefore be treated as an active mechanism in models of doctoral mental health. It shapes what counts as success or failure, how age is mobilized or silenced, and which emotional responses are perceived as legitimate.

### Age as life-course project in shi-men

Life-course perspectives emphasize that adults evaluate educational trajectories against shared norms about “on-time” and “off-time” transitions (Phan, 2024). Our findings show how such norms are intensified in Chinese doctoral settings. Older students evaluated their progress against powerful scripts about “wasting youth,” age-limited job opportunities and obligations to marry or have children by certain ages. When these scripts intersected with competitive shi-men cultures, relatively ordinary academic setbacks could be experienced as threats to the entire life project.

Younger students were also shaped by age norms. In supportive–competitive groups, they felt responsible for converting their “youth advantage” into rapid output. In laissez-faire—loose groups, they feared “wasting time” in unstructured drift. Across age groups, the lack of explicit discussion about age from supervisors meant that age operated as a quasi-hidden curriculum: students constantly compared themselves with same-age peers inside and beyond academia, whereas institutions rarely acknowledged age as a meaningful dimension of inequality. This suggests that the high prevalence of distress among PhD students (Satinsky et al., 2021) cannot be fully understood without attending to life-course structures and to how age norms are enacted in specific supervisory cultures.

### Supervisory responses to age: buffering, intensifying and silencing

Our micro-interactional analysis of how supervisors responded when age surfaced—by legitimizing delay, urging acceleration or remaining silent—adds texture to existing research on supervision and mental health (Hazell et al., 2024; Mavrogalou-Foti et al., 2024). When supervisors explicitly legitimized slower progress and reframed value beyond age (“your value is not your age but the quality of your work”), they temporarily loosened the coupling between stress and self-criticism. Students felt recognized as people, not just as delayed timelines, and could re-author their trajectories as non-standard yet legitimate. However, in competitive groups where early completion and high publication counts remained central status markers, these buffering effects were fragile.

Conversely, age-based urging (“you are not young; if you do not graduate next year, no one will hire you”) sometimes enhanced focus. More often, it intensified the burnout–self-attack–depressive mood sequence for older students already stretched by multiple roles. Age became a weapon in the internal monolog, reinforcing exactly the self-criticism that He and Mu (2025) identify as a key mediator of depressive symptoms.

Supervisory silence—especially in laissez-faire–loose groups—left age anxieties unacknowledged and unprocessed. Older students interpreted this silence as a signal that their worries were illegitimate or shameful. Younger students took it to mean that age “should not matter” and thus blamed themselves for caring about timelines at all. In both cases, age anxiety escalated privately, often compounded by teasing or subtle status hierarchies among peers. Taken together, these patterns suggest that age-sensitive supervision cannot be reduced to offering generic empathy. Supervisors need conceptual and practical tools to talk about age and life-course pressures in ways that neither catastrophise nor trivialize students’ concerns.

### Implications for practice and policy

Our findings point to several practical implications for doctoral education. First, interventions should be tailored to supervisor–group ecologies rather than delivered as generic wellbeing training. In supportive–competitive shi-men, professional development can focus on how to maintain high standards while reducing harmful comparison—e.g., shifting from rank-order evaluation to criterion-based feedback, recognizing heterogeneous timelines for younger and older students, and avoiding instrumental use of age as a threat. In laissez-faire–loose groups, training should address the risks of “benign neglect” by helping supervisors establish clear, collaboratively negotiated milestones, provide predictable feedback cycles, and check in proactively—especially with younger students who lack prior research experience.

Second, institutions should scrutinize age-related rules and informal norms that amplify age anxiety, such as rigid completion deadlines, stigma around delayed graduation, and age caps in recruitment. Transparent and flexible policies on extensions, part-time study, and non-linear trajectories would directly address mechanisms identified in our data and align with calls for multilevel resource provision in doctoral programs (Acharya and Rajendran, 2023). At a minimum, institutions can make completion rules legible, reduce uncertainty about “acceptable” timelines, and ensure that students are not penalized for requesting extensions or adjusting pace due to caregiving, health, or employment demands.

Third, student-facing services should be designed around differentiated stress–burnout configurations. Younger students in competitive groups may benefit from support targeting perfectionism and social comparison; older students juggling family and work may need structured opportunities to address life-course tensions and career uncertainty; and students in loose groups may require academic mentoring in addition to counseling. One-size-fits-all “wellbeing workshops” are unlikely to reach these distinct constellations of risk.

To translate these implications into action, we propose an age-sensitive supervision “toolkit” oriented to three stakeholder groups:

*Supervisors:* (i) initiate a normalizing conversation about timelines at key transition points (entry, candidacy, pre-submission); (ii) respond to “age anxiety” with validation plus planning (e.g., scenario mapping and milestone negotiation) rather than dismissal or catastrophizing; (iii) reduce group-level comparison triggers by making expectations explicit, diversifying recognition beyond publication counts, and discouraging teasing or status hierarchies tied to age.

*Programs/Institutions:* (i) standardize transparent milestone expectations while permitting justified pacing differences; (ii) provide structured mentoring options for students in loosely organized groups (e.g., secondary mentors, peer mentoring); (iii) strengthen career services that explicitly address age-graded opportunity structures and multiple pathways beyond a single “on-time” trajectory.

*Student support services:* (i) triage support by ecology (competitive vs. loose) and life-course constraints (caregiving/work); (ii) integrate academic skills support with psychological support where supervision is weak; (iii) offer targeted groups or coaching on social comparison, self-criticism, and coping with timeline pressures.

### Limitations and future directions

This study has limitations. Our sample is confined to 28 students in three research-intensive Chinese universities. Experiences in other institution types and regions may differ. As in most qualitative research, our aim was analytic generalization rather than statistical representativeness (Maxwell, 2012). Data are cross-sectional and retrospective. Longitudinal work could trace how stress–burnout trajectories and age meanings evolve over the course of a doctorate.

We also relied solely on student accounts, so supervisors’ perspectives on age, timelines and responsibility remain unexplored. Future research could adopt multi-perspectival and mixed-methods designs that combine qualitative typologies of supervisor–group culture with survey measures of wellbeing (Nagy et al., 2019; Mavrogalou-Foti et al., 2024). In addition, while gender and discipline are reported in Appendix 3, our analytic focus was not to compare subgroups on these dimensions. We nevertheless observed that several accounts connected age-related anxiety to life-course expectations that are often gendered in practice (e.g., family planning and caregiving responsibilities), suggesting potentially important intersections between age, gendered norms, and distress. We also noted that both supervisor–group ecologies were described across STEM and non-STEM disciplines, indicating that the typology is not discipline-specific; however, disciplinary work organization and evaluative regimes (e.g., lab-based collective work and rapid publication expectations versus more individualized research and writing trajectories) may shape how peer comparison, supervision, and age identities are negotiated. Future research with purposive stratification across gender and disciplines could examine these intersections more directly.

## Conclusion

Despite these limitations, this study contributes a more relational and context-sensitive understanding of age-moderated stress–burnout mechanisms among PhD students. It shows that age is not a neutral covariate but a life-course project negotiated within shi-men “academic families” and structured by supervisor–group cultures and wider age norms. By connecting individual-level psychological models (He and Mu, 2025) with ecological accounts of supervisory and institutional resources (Acharya and Rajendran, 2023), this study underscores that any serious attempt to address doctoral mental health—whether in China or elsewhere—must attend to how students “do age” within the specific supervisory ecologies that organize their everyday academic lives.

## Data Availability

The raw data supporting the conclusions of this article will be made available by the authors, without undue reservation.
