# Supplementary material for: Supervisor–group culture, age and the stress–burnout mechanism: a qualitative study of Chinese doctoral students
Source: Front Psychol. 2026 Mar 3;17:1794711. doi: 10.3389/fpsyg.2026.1794711 (PMC12992317; doi:10.3389/fpsyg.2026.1794711)
Supplement: Supplementary file 1 [file Table_1.docx]

**Appendix 1. Semi-structured interview guide (abbreviated)**

***Section 1: Background and doctoral trajectory***

Can you tell me a little about yourself and your doctoral journey so far?

How old are you now, and how old were you when you started the PhD?

What were your main reasons for starting a PhD at that particular time in your life?

***Section 2: Supervisor relationship***

How would you describe your relationship with your supervisor?

In what ways does your supervisor support your work and development?

In what ways, if any, does your supervisor put pressure on you (e.g., deadlines, publications)?

***Section 3: Research group/shi men culture***

Who is considered part of your shi men or research group?

How would you describe the atmosphere in the group (competitive, collaborative, loose, etc.)?

How do you feel you fit into this culture? Does your age affect this in any way?

***Section 4: Academic stress, burnout and self-criticism***

Can you describe a recent period when you felt particularly stressed about your PhD?

What happened as that stress continued—did you feel exhausted, detached, or “burnt out”?

When things go badly, how do you tend to talk to yourself? Do you become self-critical?

***Section 5: Mood and mental health***

Have there been times during your PhD when you felt low, hopeless or very discouraged?

How did those feelings relate to your work, your supervisor and your research group?

Did you talk to anyone about these feelings? Why or why not?

***Section 6: Age, “age anxiety” and comparison***

How important is age to you when you think about your PhD and future plans?

Do you compare yourself with peers of similar or different ages (inside or outside the PhD)?

Have you experienced what you would call “age anxiety”? What triggered it?

***Section 7: Supervisor–group responses to age-related concerns***

Have you ever talked with your supervisor about age-related worries (e.g., timing of graduation, job, family)?

How did your supervisor respond (e.g., encouraging extension, pushing for faster progress, avoiding the topic)?

How did group members (senior students, peers) respond to your age-related concerns?

***Section 8: Coping, resources and suggestions***

When you feel stressed, burnt out or anxious about age, what helps you cope?

What could supervisors and research groups do differently to support students of different ages?

If you could redesign doctoral policies to be more “age-sensitive”, what would you change?

**Appendix 2. Abbreviated coding manual (code families and definitions)**

***1. Academic stress and demands***

Definition: Perceived pressures and demands related to doctoral work.

Example sub-codes: publication pressure; funding insecurity; project overload; unclear expectations.

***2. Burnout and disengagement***

Definition: Experiences of emotional exhaustion, cynicism, or withdrawal from work.

Example sub-codes: emotional exhaustion; loss of interest; procrastination; physical fatigue.

***3. Self-criticism and self-evaluation***

Definition: Harsh self-judgements and negative evaluations of one’s competence or worth.

Example sub-codes: “not good enough” talk; perfectionistic standards; shame after setbacks.

***4. Depressive mood and hopelessness***

Definition: Narratives of low mood, hopelessness or loss of meaning linked to the PhD.

Example sub-codes: feeling stuck; thoughts of quitting; sense of “wasting youth”.

***5. Supervisor practices***

Definition: Specific behaviours and interaction patterns attributed to the supervisor.

Example sub-codes: academic guidance; emotional support; monitoring and control; neglect/absence.

***6. Group norms and shi men culture***

Definition: Shared expectations, values and informal rules within the research group.

Example sub-codes: competition vs cooperation; loyalty and obedience; comparison and ranking; group solidarity.

***7. Age salience and age-related comparisons***

Definition: Moments when age becomes explicit in participants’ interpretations.

Example sub-codes: being “too old/too young”; being off-time; comparing with same-age peers outside academia; comparing with younger lab mates.

***8. Age anxiety and temporal pressure***

Definition: Worries about time, age and life-course timing.

Example sub-codes: fear of “running out of time”; family/childbearing concerns; employment age limits; pressure to finish quickly.

***9. Responses to age-related concerns***

Definition: How supervisors and group members respond when age becomes an issue.

Example sub-codes: legitimising extensions; pushing for timely completion; normalising delay; stigmatising late graduation.

***10. Coping strategies and resources***

Definition: Ways in which students manage stress, burnout and age anxiety.

Example sub-codes: seeking social support; self-care practices; cognitive reframing; withdrawal/avoidance; use of institutional services.

**Appendix 3 Overview of interview participants (pseudonymised)**

All names and institutional identifiers are pseudonyms. Ages are at the time of interview.

| **ID** | **Pseudonym** | **Age** | **Age group** | **Gender** | **Discipline** | **University** | **Supervisor–group configuration** |
| --- | --- | --- | --- | --- | --- | --- | --- |
| P01 | Ying | 27 | Younger | Female | STEM | University A | Supportive–competitive |
| P02 | Wei | 25 | Younger | Male | STEM | University A | Supportive–competitive |
| P03 | Lin | 26 | Younger | Female | Humanities | University B | Supportive–competitive |
| P04 | Kai | 24 | Younger | Male | STEM | University A | Supportive–competitive |
| P05 | Lian | 25 | Younger | Female | Humanities | University B | Supportive–competitive |
| P06 | Ming | 27 | Younger | Male | STEM | University C | Supportive–competitive |
| P07 | Rui | 28 | Younger | Female | STEM | University C | Supportive–competitive |
| P08 | Bo | 26 | Younger | Male | Social sciences | University B | Laissez-faire–loose |
| P09 | An | 25 | Younger | Female | Social sciences | University B | Laissez-faire–loose |
| P10 | Qiao | 24 | Younger | Female | STEM | University A | Laissez-faire–loose |
| P11 | Tao | 27 | Younger | Male | STEM | University C | Laissez-faire–loose |
| P12 | Ning | 26 | Younger | Female | Humanities | University C | Laissez-faire–loose |
| P13 | Jie | 28 | Younger | Male | Social sciences | University A | Laissez-faire–loose |
| P14 | Xue | 25 | Younger | Female | STEM | University B | Laissez-faire–loose |
| P15 | Hui | 33 | Older | Female | STEM | University A | Supportive–competitive |
| P16 | Chen | 35 | Older | Male | Social sciences | University B | Supportive–competitive |
| P17 | Zhao | 31 | Older | Male | STEM | University C | Supportive–competitive |
| P18 | Yan | 32 | Older | Female | Social sciences | University B | Supportive–competitive |
| P19 | Dong | 37 | Older | Male | STEM | University A | Supportive–competitive |
| P20 | Shu | 34 | Older | Female | Humanities | University C | Supportive–competitive |
| P21 | Rong | 30 | Older | Female | STEM | University C | Supportive–competitive |
| P22 | Li | 32 | Older | Male | Humanities | University B | Laissez-faire–loose |
| P23 | Mei | 34 | Older | Female | Social sciences | University C | Laissez-faire–loose |
| P24 | Qin | 36 | Older | Male | STEM | University A | Laissez-faire–loose |
| P25 | Fang | 31 | Older | Female | Social sciences | University B | Laissez-faire–loose |
| P26 | Jian | 30 | Older | Male | STEM | University C | Laissez-faire–loose |
| P27 | Lan | 33 | Older | Female | Social sciences | University A | Laissez-faire–loose |
| P28 | Gu | 37 | Older | Male | STEM | University B | Laissez-faire–loose |
